# Complete Genome Sequence of *Corynebacterium falsenii* DSM 44353 To Study the Evolution of *Corynebacterium* Cluster 3 Species

**DOI:** 10.1128/genomeA.00158-14

**Published:** 2014-03-06

**Authors:** Alina Glaub, Christina Bomholt, Kerstin Gravermann, Karina Brinkrolf, Andreas Albersmeier, Christian Rückert, Andreas Tauch

**Affiliations:** Institut für Genomforschung und Systembiologie, Centrum für Biotechnologie, Universität Bielefeld, Bielefeld, Germany

## Abstract

*Corynebacterium falsenii* is a member of the natural microflora of wild and domesticated birds and is rarely detected in human clinical specimens. The chromosomal sequence of the type strain *C. falsenii* DSM 44353 comprises 2,677,607 bp and provides detailed insights into the evolution of *Corynebacterium* species assigned to the highly diverse cluster 3.

## GENOME ANNOUNCEMENT

*Corynebacterium falsenii* was first described by Sjödén et al. (1) in a polyphasic taxonomic study of four coryneform strains that were isolated from human blood cultures and a cerebrospinal fluid sample between 1991 and 1995. Based on the alignments of *rpoB* gene sequences, *C. falsenii* was grouped into the cluster 3 of the genus *Corynebacterium*, with *Corynebacterium jeikeium* being the closest phylogenetic relative (2). The species *C. falsenii* is represented by the type strain DSM 44353, which was isolated in 1994 from a blood culture of a 4-year-old boy suffering from acute lymphatic leukemia (1). However, the clinical significance of *C. falsenii* remains largely unknown, as it has since been recovered very rarely from human clinical material (3, 4). On the other hand, *C. falsenii* was isolated from the respiratory tracts of eagles and black storks (5, 6) and from bioaerosols sampled in duck houses (7). *C. falsenii* was also detected by 16S rRNA gene sequencing in the cloacal microbial community of black-winged stilts (8). Hence, *C. falsenii* may represent a member of the natural microflora of wild and domesticated birds.

To get insights into the genetic organization of this rarely recovered corynebacterium, we sequenced the genome of the *C. falsenii* type strain (1). *C. falsenii* DSM 44353 (BL 8171, CCUG 33651) was obtained from the Leibniz Institute DSMZ (Braunschweig) and grown in brain heart infusion broth-yeast extract at 37°C (9). Genomic DNA was purified with the Genomic-tip 500/G system and the Genomic DNA buffer set (Qiagen), and it was used as starting material to prepare a standard sequencing library according to the workflow of the Nextera DNA sample preparation kit (Illumina). The genomic library was sequenced in a 2 × 250 nucleotide (nt) paired-end run using the MiSeq reagent kit version 2 and the MiSeq desktop sequencer (Illumina), resulting in 1,077,270 reads and an 81-fold genome coverage. The reads were preprocessed by quality trimming in such a way that the terminal five nucleotides had a Phred quality value of ≥30 (10). Preprocessed reads were assembled with the GS *de novo* assembler software (release 2.8) to yield 44 contigs in 12 scaffolds. The software r2cat (11) supported the ordering of the scaffolds according to alignments with the chromosomal sequence of *C. jeikeium* K411 (12). The remaining gaps in the genome sequence were closed *in silico* with the Consed software (version 24) (13).

The genome sequence of *C. falsenii* DSM 44353 includes a circular chromosome of 2,677,607 bp (63.18% G+C content) and the circular corynephage ΦCFAL8171I genome of 42,009 bp (61.74% G+C content). An identical linear copy of ΦCFAL8171I is present in the chromosome as a prophage, suggesting that this corynephage had entered a lytic cycle in a subpopulation of the culture used to prepare the genomic DNA. The automatic annotation of the genome sequence with the NCBI Prokaryotic Genome Annotation Pipeline and the GeneMarkS+ software (version 2.3) revealed 2,248 protein-coding regions, 35 pseudogenes, 50 tRNA genes, 1 noncoding RNA (ncRNA) gene, and 3 rRNA operons in the chromosome of *C. falsenii* DSM 44353 and 58 protein-coding regions in the circular genome of ΦCFAL8171I.

### Nucleotide sequence accession numbers.

This genome project has been deposited in the GenBank database under accession no. CP007156 (chromosome) and CP007157 (ΦCFAL8171I).

## References

[1] Sjödén B Funke G Izquierdo A Akervall E Collins MD 1998 Description of some coryneform bacteria isolated from human clinical specimens as *Corynebacterium falsenii* sp. nov. Int. J. Syst. Bacteriol. 48(Pt 1):69–74. 10.1099/00207713-48-1-69 9542078

[2] Khamis A Raoult D La Scola B 2004 *rpoB* gene sequencing for identification of *Corynebacterium* species. J. Clin. Microbiol. 42:3925–3931. 10.1128/JCM.42.9.3925-3931.2004 15364970 PMC516356

[3] Bernard KA Munro C Wiebe D Ongsansoy E 2002 Characteristics of rare or recently described *Corynebacterium* species recovered from human clinical material in Canada. J. Clin. Microbiol. 40:4375–4381. 10.1128/JCM.40.11.4375-4381.2002 12409436 PMC139690

[4] Iroh Tam PY Fisher MA Miller NS 2010 *Corynebacterium falsenii* bacteremia occurring in an infant on vancomycin therapy. J. Clin. Microbiol. 48:3440–3442. 10.1128/JCM.00990-10 20610679 PMC2937743

[5] Fernández-Garayzábal JF Egido R Vela AI Briones V Collins MD Mateos A Hutson RA Domínguez L Goyache J 2003 Isolation of *Corynebacterium falsenii* and description of *Corynebacterium aquilae* sp. nov., from eagles. Int. J. Syst. Evol. Microbiol. 53:1135–1138. 10.1099/ijs.0.02533-0 12892140

[6] Fernández-Garayzábal JF Vela AI Egido R Hutson RA Lanzarot MP Fernández-García M Collins MD 2004 *Corynebacterium ciconiae* sp. nov., isolated from the trachea of black storks (*Ciconia nigra*). Int. J. Syst. Evol. Microbiol. 54:2191–2195. 10.1099/ijs.0.63165-0 15545457

[7] Martin E Kämpfer P Jäckel U 2010 Quantification and identification of culturable airborne bacteria from duck houses. Ann. Occup. Hyg. 54:217–227. 10.1093/annhyg/mep088 20042465

[8] Santos SS Pardal S Proença DN Lopes RJ Ramos JA Mendes L Morais PV 2012 Diversity of cloacal microbial community in migratory shorebirds that use the Tagus estuary as stopover habitat and their potential to harbor and disperse pathogenic microorganisms. FEMS Microbiol. Ecol. 82:63–74. 10.1111/j.1574-6941.2012.01407.x 22571242

[9] Bomholt C Glaub A Gravermann K Albersmeier A Brinkrolf K Rückert C Tauch A 2013 Whole-genome sequence of the clinical strain *Corynebacterium argentoratense* DSM 44202, isolated from a human throat specimen. Genome Announc. 1(5):e00793-13. 10.1128/genomeA.00793-13 24092787 PMC3790091

[10] Ewing B Green P 1998 Base-calling of automated sequencer traces using Phred. II. Error Probabilities. Genome Res. 8:186–194 9521922

[11] Husemann P Stoye J 2010 r2cat: synteny plots and comparative assembly. Bioinformatics 26:570–571. 10.1093/bioinformatics/btp690 20015948 PMC2820676

[12] Tauch A Kaiser O Hain T Goesmann A Weisshaar B Albersmeier A Bekel T Bischoff N Brune I Chakraborty T Kalinowski J Meyer F Rupp O Schneiker S Viehoever P Pühler A 2005 Complete genome sequence and analysis of the multiresistant nosocomial pathogen *Corynebacterium jeikeium* K411, a lipid-requiring bacterium of the human skin flora. J. Bacteriol. 187:4671–4682. 10.1128/JB.187.13.4671-4682.2005 15968079 PMC1151758

[13] Gordon D Abajian C Green P 1998 Consed: a graphical tool for sequence finishing. Genome Res. 8:195–202. 10.1101/gr.8.3.195 9521923

